# Molecular epidemiology of respiratory syncytial virus in children with acute respiratory illnesses in Africa: A systematic review and meta-analysis

**DOI:** 10.7189/jogh.13.04001

**Published:** 2023-01-14

**Authors:** Belay Tafa Regassa, Lami Abebe Gebrewold, Wagi Tosisa Mekuria, Nega Assefa Kassa

**Affiliations:** 1 Department of Medical Laboratory Sciences, College of Medicine and Health Sciences, Ambo University, Ethiopia; 2 Department of Public Health, College of Medicine and Health Sciences, Ambo University, Ethiopia; 3 School of Public Health, College of Health and Medical Sciences, Haramaya University, Ethiopia

## Abstract

**Background:**

Globally, the respiratory syncytial virus (RSV) is the most common etiologic agent of acute respiratory illnesses in children. However, its burden has not been well addressed in developing countries. We aimed to estimate the molecular epidemiology of RSV in children less than 18 years of age with acute respiratory infections in Africa by conducting a systematic review and meta-analysis.

**Methods:**

We systematically searched PubMed, Scopus, CINAHL, and Global Index Medicus databases to identify studies published from January 1, 2002, to April 27, 2022, following the PRISMA 2020 guideline. We assessed the study quality using the Joanna Brigg’s Institute (JBI) critical appraisal checklists. We conducted a qualitative synthesis by describing the characteristics of included studies and performed the quantitative synthesis with random effects model using STATA-14. We checked for heterogeneity with Q statistics, quantified by *I*^2^, and determined the prediction interval. We performed subgroup analyses to explain the sources of heterogeneity and assessed publication biases by funnel plots augmented with Egger’s test.

**Results:**

Eighty-eight studies with 105 139 participants were included in the review. The overall pooled prevalence of RSV in children <18 years of age was 23% (95% confidence interval (CI) = 20, 25%). Considerable heterogeneity was present across the included studies. The adjusted prediction interval was found to be 19%-27%. Heterogeneities were explained by subgroups analyses. The highest prevalence of RSV was found among inpatients, 28% (95% CI = 25, 31%) compared with inpatients/outpatients and outpatients, with statistically significant differences (*P* < 0.01). The RSV estimate was also highest among those with acute lower respiratory tract illnesses (ALRTIs), 28% (95% CI = 25, 31%) compared with acute upper respiratory tract illnesses (AURTIs) and both acute upper/lower respiratory manifestations, with statistically different prevalence (*P* < 0.01). RSV infection estimates in each sub-region of Africa were statistically different (*P* < 0.01). There were no statistically significant differences in RSV infections by designs, specimen types, and specimen conditions, despite them contributing to heterogeneity.

**Conclusions:**

We found a high prevalence of RSV in pediatric populations with acute respiratory tract illnesses in Africa, highlighting that the prevention and control of RSV infections in children deserve more attention.

**Registration:**

PROSPERO CRD42022327054

Acute respiratory tract infections caused by a range of respiratory viruses are linked to a spectrum of upper and lower respiratory tract syndromes [1,2]. The burden of acute respiratory infections is increasing as novel respiratory pathogens are being discovered by molecular methods [3]. Consequently, over 200 different virus types have been found to cause acute respiratory infections, with the respiratory syncytial virus (RSV), influenza viruses, human metapneumovirus, parainfluenza viruses, adenoviruses, rhinoviruses, coronaviruses, and bocaviruses being the most prevalent [4].

RSV is a single-stranded ribonucleic acid (RNA) virus affecting respiratory epithelial cells; it has two subtypes, RSV-A and RSV-B, with antigenic differences. These differences are found in the attachment glycoprotein G, and the RSV G protein shows the highest degree of divergence, both between and within the two groups [5,6].

RSV is one of the most common etiologic agents of acute respiratory infections causing severe disease and mortality, particularly in very young children, but also in other age groups and in at-risk groups [7]. Globally, the virus has led to 33 million episodes of acute lower respiratory tract infection, 3.6 million hospital admissions, 26 300 in-hospital deaths, and 101 400 RSV-attributable overall deaths in children younger than five years [8]. RSV is also the most prevalent cause of severe lower respiratory tract infection in the first six months of life. Most deaths occur in low- and middle-income countries [8,9]. Ninety-nine percent (99%) of these deaths occurred in developing countries, though the actual mortality rate due to RSV infection is higher than reported [10,11].

Currently, ribavirin is the only one licensed antiviral medication for treating RSV infection; it has a very limited efficacy and high toxicity. Its use is usually reserved for severely immunocompromised children. To date, only the maternal antibody palivizumab is available for preventing RSV infection; it has been shown to reduce hospital admission due to RSV infection in some high-risk infants. There are several RSV vaccines under development or undergoing clinical trials in humans. Understanding the disease burden caused by RSV will facilitate the study of the effectiveness of antivirals and vaccines [12].

Generally, approaches for detecting RSV have not been widely accessible [13] and its exact burden in different geographical regions has not been fully determined, despite evidence that RSV was the most widely detected virus in children [14]. Lack of data on disease burden is especially problematic in developing countries with high numbers of severe cases and deaths, as the true burden of the disease can only be estimated with adequate epidemiological data on RSV infections in different settings [15]. For these reasons, we conducted a systematic review and meta-analysis on molecular epidemiology of RSV in children under 18 years of age with acute respiratory infections in Africa.

## METHODS

### Databases and search strategies

We registered the review protocol in the International Prospective Register of Systematic Reviews (PROSPERO, registration number CRD42022327054). We comprehensively searched PubMed, Scopus, CINAHL, and Global Index Medicus (GIM) databases with keywords, medical subject headings (MeSH), and related terms (full search strategy is available in Appendix S1 in the **Online Supplementary Document**). We also searched for gray literature through Mednar, worldwide science, and Grey Literature Report. Additionally, we searched articles from reference lists of included studies and related systematic reviews and meta-analyses through Google scholar and African Journal Online (AJOL).

### Inclusion and exclusion criteria

We screened titles and abstracts and evaluate full texts for eligibility based on pre-defined inclusion and exclusion criteria.

#### Inclusion criteria

We included observational studies (including cross-sectional, case-control, and cohort studies) and studies addressing the molecular epidemiology of respiratory syncytial virus in children less than 18 years of age reporting RSV positive cases and/or RSV subgroups from Africa. Children less than 18 years of age with clinical diagnosis of acute respiratory tract infections (acute upper respiratory tract infection and/or acute lower respiratory tract infections) were included, while only considering reverse transcriptase-polymerase chain reaction (RT-PCR) diagnosed/confirmed RSV cases (including rapid molecular tests, multiplex molecular tests, and conventional PCR of respiratory specimens). We included only studies conducted in Africa and published in the English language between January 01, 2002, and April 27, 2022.

### Exclusion criteria

We excluded original studies performed outside of Africa, review papers, books, letters, brief report, case reports, meeting reports, poster presentations, and studies published before January 01, 2002, during the screening of titles and abstracts, while articles with irretrievable full texts, records with unrelated outcome measures, or articles with missing outcomes were excluded during full text screening. We excluded RSV cases identified through viral culture, rapid antigen detection tests, or direct immunofluorescent antibody tests and studies of asymptomatic RSV infections.

## Study selection and quality assessment

We imported all retrieved studies to Endnote X9 and deduplicated, after which two reviewers (BTR and LAG) independently screened all their titles and abstracts for eligibility according to inclusion and exclusion criteria. Discrepancies were discussed and until a consensus was reach and the full text was accessed if necessary. Then, the two reviewers assessed the full texts of the remaining articles for eligibility. After this step, two investigators assessed the study quality independently using the Joanna Brigg’s Institute (JBI) critical appraisal checklist adapted for observational studies (checklist for cross-sectional studies, case-control studies, and cohort studies containing 8, 10 and 11 items, respectively). If discrepancies in the rating occurred, the two reviewers discussed and resolved the issues. If the issue was not resolved through discussion, the third reviewer’s decision was accepted. Accordingly, studies with the number of positive responses (yes) greater than or equal to half (≥50%) of the number of checklist items relevant for the specific study were included in the systematic review and meta-analysis.

## Data extraction and management

Data extractions were performed on excel spreadsheet containing the following items: study first author and publication year, study population, number of cases positive for RSV, sample size, RSV subgroups, clinical manifestations (upper respiratory illness, and/or lower respiratory illness), patient categories (inpatients, outpatients or both), study design, sampling method, specimen type, specimen condition (stored or fresh), study setting (rural, urban or both), country, and sub-region of Africa (Table S1 in the **Online Supplementary Document**). Extractions were done by two independent reviewers. Where results were published multiple times, the data was used only once. Uncertainties during the extraction process were resolved by joint discussion between the reviewers.

## Strategy for data synthesis

We followed the Preferred Reporting Items for Systematic reviews and Meta-Analyses (PRISMA) 2020 guidelines [16]. We used a flow diagram to illustrate the literature search and article selection processes and a table to provide an overview of the included articles’ characteristics, which we described using a qualitative synthesis performed with the STATA software (Version 14.0, StataCorp, Texas, USA). A random effects model was also applied. We checked for heterogeneity across studies and performed subgroup analyses for age groups, patient categories, clinical manifestations, sub-regions of Africa, study designs, specimen types as well as specimen conditions to identify the sources of heterogeneity. Heterogeneity was evaluated by the χ^2^ test on Cochrane’s Q statistic, which was quantified by *I*^2^ values. The *I*^2^ statistic estimates the percentage of total variation across studies due to true between-study differences rather than chance. We used comprehensive meta-analysis (CMA) prediction intervals program (Biostat Inc., New Jersey, USA) to determine the adjusted prediction interval to indicate how much the effect sizes vary in 95% of all comparable populations. By omitting one study at a time using STATA software, we conducted a sensitivity analysis to check the stability of summary estimate. We assessed publication biases by visually inspecting funnel plots, augmented with statistical testing using the Egger’s test.

## RESULTS

### Review processes and findings

**Figure 1:**
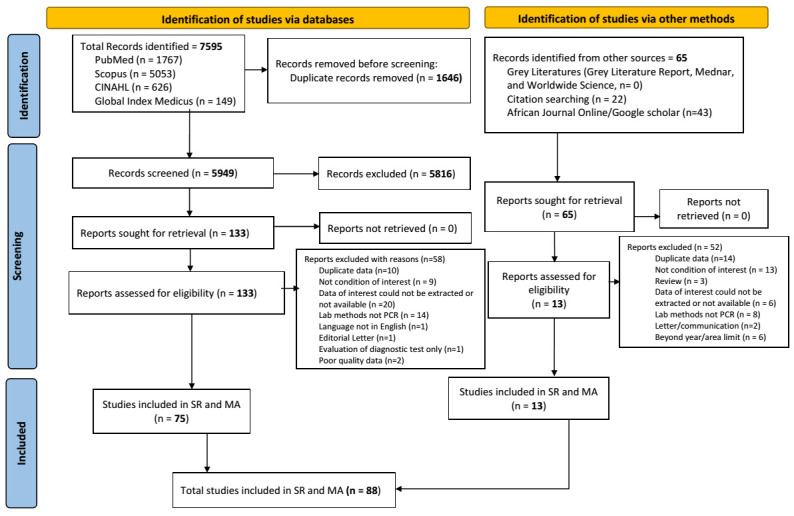
PRISMA 2020 flow diagram for literature search and selection.

We retrieved a total of 7595 articles from the databases (PubMed = 1767, Scopus = 5053, CINAHL = 626, Global Index Medicus = 149), limiting our search to the period from January 1, 2002, to April 27, 2022. After deduplication (n = 1646) using Endnote, 5949 records remained. We then screened the titles and abstracts and removed 5816 irrelevant studies. Of the 133 full texts retrieved and assessed for eligibility, we excluded 58 and retained 75 as relevant. Additionally, records were sought from other sources such as free web search engines (African Journal Online and Google Scholar) and Grey Literature, from which we retrieved 65 full text records, 52 of which were not eligible and 13 were found retained and included. At the end, 88 articles were found to be relevant for this systematic review and meta-analysis (**Figure 1**).

### Characteristics of the included studies

Eighty-eight studies with 105 139 study participants from 25 African countries were included in this systematic review and meta-analysis. JBI quality assessment tools were applied and all included studies satisfied the required level of methodological quality. The minimum and maximum sample sizes utilized were 51 [17] and 9969 [18], respectively. Considering the number of studies included from sub-regions of Africa, 24 reports were from Northern, 23 from Eastern, 18 from Southern, 16 from Western, and six from Middle Africa, while one study presented data from three African sub-regions (**Table 1**).

**Table 1:** Studies included in SR and MA by country and sub-regions of Africa

African sub-regions	Countries	Number of studies included
**Northern Africa**	Algeria	1
	Egypt	17
	Morocco	3
	Sudan	1
	Tunisia	2
	Total	24
**Eastern Africa**	Kenya	12
	Madagascar	5
	Malawi	1
	Mozambique	2
	Tanzania	1
	Zambia	2
	Total	23
**Southern Africa**	South Africa	17
	Botswana	1
	Total	18
**Western Africa**	Burkina Faso	1
	Côte d'Ivoire	2
	Gambia	2
	Ghana	3
	Mali	2
	Niger	2
	Nigeria	2
	Senegal	2
	Total	16
**Middle Africa**	Cameroon	2
	Central African Republic	2
	DR Congo	1
	Gabon	1
	Total	6
	*Ghana, Gabon, Tanzania, Burkina Faso	1

*Research reported from multi-countries of different sub-regions.

Referring to the specific countries, 17 studies were reported from Egypt, 17 from South Africa, 12 from Kenya, five from Madagascar, three from Ghana, and three from Morocco. The remaining 22 studies were reported from the 11 other countries, with two studies from each. Eight studies were reported from eight countries, with one study reported from each, while one study was reported from four African countries (**Table 1**).

Most included studies (n = 73 (82.95%)) used a cross-sectional study design, while the rest were case-control (n = 11 (12.50%)) and cohort studies (n = 4, (4.55%)). Most studies used consecutive sampling methods with prospective time courses. Regarding the study population, two thirds of the included studies (n = 60 (68.2%)) were done on under five children or presented accessible data of the under five children; the remaining 28 studies were performed on children less than 18 years of age or presented data for the group. Approximately one third of the included studies (35.23%) described the study settings described as rural, urban, or both. Studies indicated that single types or combined respiratory specimens were taken where nasopharyngeal swabs were most utilized. All the respiratory specimens in the included studies were analyzed by reverse transcriptase PCR. All the studies described the conditions of specimens analyzed as fresh or stored, except for three studies which did not describe the specimen condition. Most included studies (n = 48 (54.55%)) had inpatients as participants. Regarding the participants’ clinical manifestations, half of the studies dealt with acute lower respiratory tract illnesses (ALRTI), while 39 (44.32%) of the studies dealt with participants with both acute upper respiratory tract illnesses (AURTI) and ALRTI. The included studies’ profiles are available in **Table 2**.

**Table 2:** Profile of studies included in the systematic review and meta-analysis

	Study profile	Articles included (n (%))
**Study design**	Cross-sectional	73 (82.95)
	Case-control	11 (12.50)
	Cohort	4 (4.55)
**Sampling method**	Consecutive	80 (90.91)
	Random	4 (4.55)
	Systematic	4 (4.55)
**Time course of study**	Prospective	76 (86.36)
	Retrospective	10 (11.36)
	Both	2 (2.27)
**Study population**	<5 y	60 (68.18)
	<18 y	28 (31.82)
**Specimen type**	NPS	29 (32.95)
	NPA	18 (20.45)
	NS	6 (6.82)
	Mixed*	28 (31.82)
	Others†	5 (5.68)
	Not described	2 (2.27)
**Specimen condition**	Fresh	43 (48.86)
	Stored	42 (47.73)
	Not described	3 (3.41)
**Study setting**	Rural	12 (13.64)
	Urban	3 (3.41)
	Rural and urban	16 (18.18)
	Not described	57 (64.77)
**Patient categories**	Inpatients	48 (54.55)
	Inpatients and outpatients	26 (29.55)
	Outpatients	10 (11.36)
	Not described	4 (4.55)
**Clinical manifestations**	ALRTI	44 (50.00)
	AURTI	5 (5.68)
	AURTI/ALRTI	39 (44.32)

NPS – nasopharyngeal swab, NPA – nasopharyngeal aspirate, NS – nasal swab, ALRTI – acute lower respiratory tract infections, AURTI – acute upper respiratory tract infections, y – years

*Nasal wash/NPS, or NS/NPS, or NPS/OPS, or NPS/NPA, or NPS/NPA/bronchoalveolar lavage.

†Bronchoalveolar lavage, oropharyngeal swab, throat swab.

### Proportions of RSV infections

The proportion of RSV infections in the studies ranged from 0.5 to 86%; the minimum was reported from the study conducted in Nigeria [19] while the highest infection proportion, 86%, was reported from Egypt [20]. The proportion of RSV for most studies (n = 28 (31.5%)) was 21%-30% [18,21-47], followed by 24 (27.3%) studies with the proportion of 11%-20% [14,17,48-69], 17 (19.3%) with 31%-50% [70-86], and 17 (19.3%) with 0.5%-10% [19,87-102]. The remaining two studies reported the highest proportions of infections, 85.3% [103] and 86% [20] (Table S1 in the **Online Supplementary Document**).

Referring to RSV sub-grouping, 25 studies reported the subgroups of RSV (Table S1 in the **Online Supplementary Document**). From the total 4095 RSV samples that underwent sub-grouping, RSV-A was identified in 2074 cases (51%), RSV-B was identified in 1812 (44%) and RSV-A and B co-infections were identified in 209 cases (5%).

### The pooled prevalence of RSV

**Figure 2:**
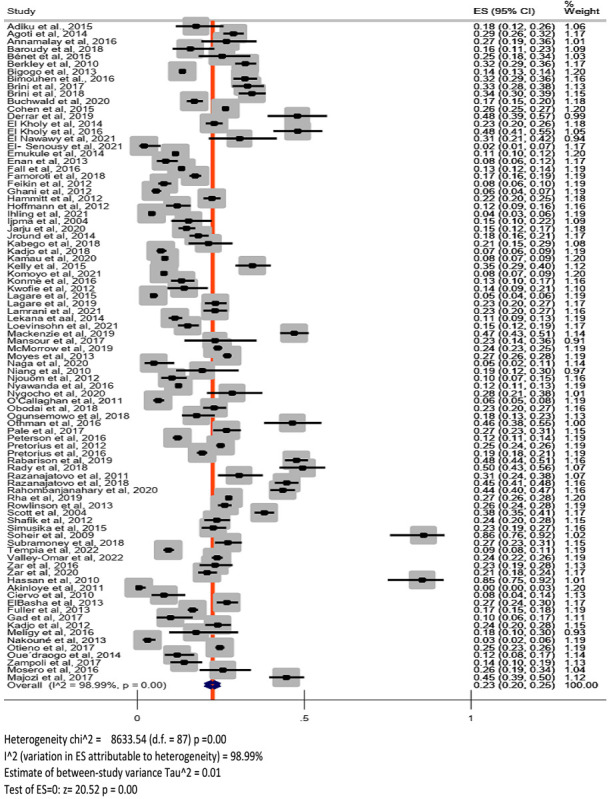
Forest plot.

The point estimates of RSV infections among children from the individual studies ranged from 0 (95% confidence interval (CI) = 0, 3%) to 86% (95% CI = 76, 92%). The overall pooled prevalence of RSV in children under 18 years of age was 23% (95% CI = 20, 25%). Considerable heterogeneity was present for the overall combined effect size (Q = 8633.54 (degrees of freedom (df) = 87), *P* < 0.01; *I*^2^ = 98.99% and *Τ*^2^ = 0.01) (**Figure 2**). The adjusted prediction interval was found to be from 19% to 27%.

By visually inspecting the forest plots, we found that some studies were outliers (**Figure 2**). Checking for the distribution of the proportions of included studies, we found that it was skewed to the right. Using a graph box, we identified two studies [20,104] as outliers. After removing them, we performed the meta-analysis and computed the pooled prevalence of RSV infections of 21% (95% CI = 19, 23%) with considerable heterogeneity (Q = 8083.69 (df = 85), *P* < 0.01; *I*^2^ = 98.95%; *T*^2^ = 0.01). The prediction interval was also found to range from 17% to 25%. We found no major difference when comparing this with the combined effect and heterogeneity statistics of the previous analysis (including all the 88 studies). We thus retained all 88 studies in the analysis.

### Subgroup analysis

The results of the subgroup analyses are presented in **Table 3****.** We found substantial heterogeneity among the individual effect sizes of the included studies, so we performed subgroups analyses to explore the potential sources of heterogeneity among them. We found that the differences in age groups, clinical manifestations, patient categories, and sub-regions of Africa most likely contributed to the heterogeneity between individual studies, as did specimen type, and specimen condition.

**Table 3:** Subgroup analyses results for the positive rate of RSV infection in children

					Heterogeneity tests			
**Groups**	**Number of studies**	**Number of RSV positive**	**Number of total participants**	**RSV positive rate (95% CI)**	**Q-value**	***P*_h_**	***I*^2^ (%)**	***P-*difference**
**Overall**	88	21 178	105 139	23% (20, 25%)	8633.54	<0.01	98.99	-
Subgroup analyses								
**Age groups**								<0.001
<5 y	60	17 154	77 219	25% (23, 28%)	5098.51	<0.01	98.84	
<18 y	28	4024	27 920	17% (13, 20%)	1917.58	<0.01	98.59	
**Patient category**								<0.001
Inpatients	48	14 086	57 453	28% (25, 31%)	3834.98	<0.01	98.77	
Inpatients and outpatients	26	5544	33 505	18% (14, 21%)	163.72	<0.01	98.95	
Outpatients	10	1401	12 239	14% (11, 17%)	2381.55	<0.01	94.50	
Not described	4	147	1942	7% (4, 10%)	19.97	<0.01	84.98	
**Clinical manifestations**								<0.001
ALRTI	44	13 578	53 795	28% (25, 31%)	3452.63	<0.01	98.75	
AURTI	5	333	1828	19% (13, 25%)	41.71	<0.01	90.41	
AURTI/ALRTI	39	7267	49 516	17% (15, 19%)	2687.58	<0.01	98.59	
Sub-regions of Africa*								<0.001
Northern	24	2313	8605	30% (24, 36%)	1131.84	<0.01	97.97	
Southern	18	10 273	42 178	23% (19, 26%)	1212.75	<0.01	98.60	
Eastern	24	6363	36 858	22% (18, 26%)	2267.36	<0.01	98.99	
Western	17	1696	11 420	17% (12, 21%)	1053.22	<0.01	98.48	
Middle	7	533	6078	9% (6, 12%)	89.24	<0.01	93.28	
**Design**								0.944
Cross-sectional	73	18 328	89 353	22% (20, 25%)	8213.42	<0.01	99.12	
Case-control	11	2401	13 641	23% (19, 27%)	340.87	<0.01	97.07	
Cohort	4	449	2145	22% (16, 28%)	36.35	<0.01	91.75	
**Specimen type**								0.625
NPS	29	5877	30 921	22% (18, 26%)	3059.09	<0.01	99.08	
NPA	18	6482	28 914	23% (18, 28%)	1625.36	<0.01	98.95	
NS	6	565	2544	16% (5, 28%)	668.64	<0.01	99.25	
Mixed†	28	7606	40 054	24% (21, 27%)	1814.32	<0.01	98.51	
Others‡	5	340	1450	17% (4, 29%)	170.32	<0.01	97.65	
Not described	2	308	1256	22% (20, 24%)	-	-	-	
**Specimen condition**								0.437
Fresh	43	11 443	57 859	21% (19, 24%)	3870.40	<0.01	98.91	
Stored	42	9038	44 087	23% (20, 27%)	4580.07	<0.01	99.10	
Not described	3	697	3193	28% (17, 39%)	-	-		

CI – confidence interval, NPS – nasopharyngeal swab, NPA – nasopharyngeal aspirate, NS – nasal swab, ALRTI – acute lower respiratory tract infections, AURTI – acute upper respiratory tract infections

*One study reported data from three sub-regions.

†Nasal wash/NPS, or NS/NPS, or NPS/OPS, or NPS/NPA, or NPS/NPA/bronchoalveolar lavage.

‡Bronchoalveolar lavage, oropharyngeal swab, throat swab.

Among the 88 studies included in the meta-analysis, 60 reported the prevalence of RSV in children under five years of age, while 28 reported the prevalence of RSV in children under 18 years age. The pooled estimates of RSV were found to be 25% (95% CI = 23, 28%) in the age group of 0-5-year-old age group and 17% (95% CI = 13, 20%) in the 0-18-year-old age group. Looking at the heterogeneity summary reported in each group, the heterogeneities were not reduced (Q = 5098.51 with df = 59, *P* < 0.01, *I*^2^ = 98.84% for children under five years, and Q = 1917.58 with df = 27, *P* < 0.01, *I*^2^ = 98.59% for children under 18 years of age). The test of group differences (Q = 18.60, (df = 1), *P* < 0.01) indicated that the group-specific overall effect sizes were statistically different.

Considering patients’ category, the prevalence of RSV was stratified into “inpatients”, “outpatients”, “inpatients/outpatients”, and “not described” categories. Looking at the heterogeneity summary reported in each group, the heterogeneities were not reduced as compared with the result of overall estimate. The highest prevalence of RSV was found among inpatients (28%; 95% CI = 25, 31%), followed by inpatients/outpatients (18%; 95% CI = 14, 21%), outpatients (14%; 95% CI = 11, 17%), and the “not described” category (7%; 95% CI = 4, 10%). The test of group differences showed that prevalence specific to patient categories were statistically different (Q = 87.90 (df = 3), *P* < 0.01).

Stratifying patients according to their respiratory syndromes (AURTI, ALRTI, and both), the highest estimate of RSV was reported among those with lower respiratory tract illnesses, 28% (95% CI = 25, 31%), with statistically different pooled prevalence between the groups (Q = 29.90 (df = 2), *P* < 0.01). We found considerable heterogeneity in each group.

The pooled prevalence of RSV was also determined by sub-regions of Africa. The highest prevalence of RSV was from Northern Africa (30%; 95% CI = 24, 36%), Southern Africa, (23%; 95% CI = 19, 26%), and Eastern Africa (22%; 95% CI = 18, 26%). The prevalence in Western and Middle Africa were 17% (95% CI = 12, 21%) and 9% (95% CI = 6, 12%), respectively. The overall estimates in each sub-region were statistically different (Q = 57.07 with df = 4, *P* < 0.01). Heterogeneity statistics also showed variations among sub-regions.

Subgroup analyses by study design, specimen type, and specimen condition also indicated that they contributed to the heterogeneity between the individual studies. However, there were no statistically significant differences in the rates of RSV infections by designs (*P* = 0.944), specimen types (*P* = 0.625), and specimen conditions (*P* = 0.437).

### Sensitivity analysis and publication bias

**Figure 3:**
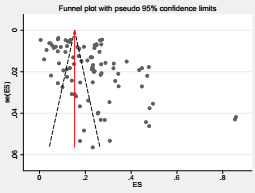
Funnel plot.

Performing the sensitivity analysis, the overall results noticeably changed after each individual study was omitted, and the combined effect was found to be 31.11 (95% CI = -4.83, 67.05%). For the overall meta-analysis of the pooled prevalence of RSV among children with acute respiratory illnesses, the funnel plot revealed evidence of publication bias (**Figure 3**). Egger’s linear regression test was performed to test for publication bias, which was detected among the publications that reported RSV positive rates for all patients (Intercept, Bo = 8.76; 95% CI = 1.48, 16.03, standard error (SE) = 3.66, *P* < 0.001).

## DISCUSSION

RSV is the most common viral cause of ARTIs in developed and developing countries [11,104]. The available epidemiological data on RSV in Africa has not been systematically summarized among the pediatric population; it requires data on the epidemiology of RSV infection in different settings to determine the disease’s true burden. We thus conducted a systematic review and meta-analysis in children under 18 years of age in Africa. After rigorous screening, 88 articles, including 105 139 patients, were deemed eligible and included in the systematic review and meta-analysis.

We found that the distribution of RSV was not uniform in the continent. Its proportion varied from 0.5 to 86% across countries. This was consistent with the previous review from Africa, which was done on all age groups [105]. This might indicate that RSV was associated with a significant burden of ARTIs in the pediatric population in Africa. The overall pooled prevalence of RSV in the current review was 23% (95% CI = 20, 25%). This result was higher than the report of the previous meta-analysis from Africa, which found the prevalence to be 14.6% (95% CI = 13.0, 16.4) [105]. Both studies had similar the methods of detecting RSV (reverse transcriptase PCR). The variation might be due to the study population, as the previous study included participants of all ages, and our study only included children. The overall positive rate of RSV infection among children from China (16.0%; 95% CI = 12.9, 19.6%) [106] was lower than the pooled prevalence in our meta-analysis. The possible reasons for the variations between the results could be geographical differences and detection methods – the study from China used both PCR and immunofluorescence assays (IFA), while we only included studies using PCR as a detection method. RSV is identified less frequently by immunofluorescent detection techniques. Compared to immunofluorescence, molecular diagnostics are more sensitive and specific [107,108]. There is evidence that the introduction of nucleic acid-based diagnostic tests has markedly improved the understanding of viral etiology among ARTI patients [109]. Therefore, the real burden of RSV in China would likely be comparable to our findings if molecular technique had been used in all included studies, though the geographical variations should also be considered.

We also found considerable heterogeneity in this meta-analysis. The Q test statistic was 8633.54 (df = 87) with *P* < 0.01, from which we found considerable variations of effect sizes between the individual studies. The *I*^2^ heterogeneity statistic also indicates the presence of heterogeneity between studies, highlighting that about 98.99% of the variability in the effect-size estimates came from the real differences between studies. This result was consistent with reports from Africa [105] and China [106] and supported by the global and national evidence that the burden of RSV varied substantially from year to year in any given population and setting [11,110]. In the current meta-analysis, the pooled prevalence was 23% (95% CI = 20, 25%). Since the *T*^2^ value was greater than zero (*T*^2^ = 0.01), the prediction interval had to be determined to indicate how much the effect sizes vary in the population of interest. We found that the adjusted prediction interval ranged from 19% to 27%, suggesting that the true effect size in 95% of all comparable populations falls in this interval.

We conducted subgroup analyses based on the age groups, clinical manifestations, patient categories, and sub-regions, to explore the potential sources of heterogeneity between the individual studies and found that these factors were the sources for the variations observed in the effect sizes.

Additionally, the subgroup analyses by study design, specimen type, and specimen condition indicated that they contributed to the heterogeneity between the individual studies. Similarly, subgroup analyses in previous studies also identified sources of heterogeneity; they were explained by age group, patient category, and sample types [106] and within all groups [105]. These variations might suggest that the burden of respiratory syncytial viral infections differs between studies from country to country, region to region, even within a county, due to several factors such as socio-demographic characteristics, geographical differences, climatic variations, and cultural differences. The heterogeneity could also result from clinical conditions of participants, methodological differences, or both.

We found the prevalence of RSV to be highest among inpatients (28%; 95% CI = 25, 31%), followed by inpatients/outpatients (18%; 95% CI = 14, 21%) and outpatients (14%; 95% CI = 11, 17%), with statistically significant differences between the groups (*P* < 0.001). This result was consistent with the results of other meta-analyses [111,112]; a higher prevalence of RSV was seen in inpatients than in outpatients. In contrast, no significant difference was reported from patient categories (inpatients and in/outpatients) in the meta-analysis from China [106]. In both developed and developing countries, RSV infections are a major cause of hospitalizations and in-hospital deaths among children [113,114]. RSV has been associated with 12%-63% of all acute respiratory infections (ARIs) and 19%-81% of viral ARIs causing hospitalization in infants and children [15]. These all indicate that RSV is the most common cause of ARIs and a major cause of hospital admissions in children, resulting in a substantial burden on health care services. There was also evidence to put the burden of RSV into this context. Compared to influenza, retrospective analyses showed that RSV causes up to 16 times more hospitalizations and emergency department visits in children aged <5 years [115-117].

We performed sensitivity analyses, and the overall results were noticeably changed after each individual study was omitted (31.11%; 95% CI = -4.83, 67.05%); this might indicate the instability of the meta-analysis results.

Funnel plot is a plot that measures the study size (usually standard error or precision) on the vertical axis as a function of effect size on the horizontal axis. In the absence of publication bias, we would expect the studies to be distributed symmetrically about the combined effect size. In the presence of bias, we would expect a higher concentration of studies on one side of the combined effect size. In this study, the funnel plot showed evidence of publication bias for the pooled prevalence of RSV in children less than 18 years of age with ARIs. Furthermore, we performed Egger’s linear regression test to check for publication bias, which was detected among the publications that reported RSV positive rates for all patients (Intercept, Bo = 8.76; 95% CI = 1.48, 16.03; SE = 3.66, *P* < 0.001). This result might show that the missing studies differ systematically from the observed studies. However, the funnel plot asymmetry may be caused by factors other than publication bias, such as a presence of a moderator correlated with the study effect and study size or, more generally, the presence of substantial between-study heterogeneity.

Our review indicated that less than one third of the included studies (n = 25 (28.4%)) performed subgroupings of RSV. From a total of 4095 RSV samples that underwent subgrouping, 51% were RSV type A, 44% were type B, and the remaining 5% were RSV type A and B co-infections. The analysis of nucleotide and amino acid sequences in detected RSV help with characterizing the pathogen’s genetic variability, tracing of its evolution, and clarification of the mechanisms required for immune escape [4]. The clinical impact of viral factors during RSV infection is still controversial, as there are conflicting reports regarding the associations of different groups and genotypes with severity of infection [118]. Regarding the relationship between virulence and RSV strains, evidence shows that specific genotypes of subtype A are related to increased illness severity [119-121]. Linking specific RSV strains to human disease severity with confidence is challenging and no consistent picture of virulent strains has yet emerged [118]. Determination of circulating RSV subgroups and genotypes in different countries is important in terms of the development of effective vaccine.

This review has some limitations. One of the concerns is that we found substantial heterogeneity in estimating the prevalence of RSV infection in children with ARI across the included studies. Consequently, some sources of heterogeneity were identified. For example, sub-regions, clinical syndromes and patient categories were the investigated potential sources of heterogeneity in our study. However, there may still be other sources of heterogeneity that have not yet been determined. We had difficulty in classifying the children’s age groups due to incommensurability of ages in these studies. Therefore, the interpretation of the results should be with cautious due to the difficulties in comparing some of these studies. Lastly, the amount of data available per country is variable with multiple studies from certain countries, which may bias the data.

Despite the above-mentioned limitations, this study has several strengths. This systematic review and meta-analysis used a predefined and registered protocol. The systematic searches performed in this review included many medical literature databases, so numerous studies were retrieved spanning a large time period. Additionally, this was complemented with a gray literature search using additional search engines. Two independent investigators were involved in all stages of the review process. Studies that identified RSV using reverse transcriptase PCR, with the highest sensitivity and specificity, were included. Sufficient data existed in the original studies to answer the objective of our review. Most of the included studies were prospective, where testing bias could not have occurred.

## CONCLUSIONS

We found a high prevalence of RSV in the pediatric population with ARTI in Africa. Consequently, the prevention and control of RSV infections in children deserves more attention from health care providers, researchers, policymakers, and stakeholders for detection, management, and efficient control. Like other infectious diseases, surveillance systems for RSV should be widely initiated or incorporated into existing surveillance programs. Furthermore, efforts to address this burden could mainly focus on primary prevention including development and implementation of vaccines against RSV.

## Additional material


Online Supplementary Document
