# Supplementary material for: Molecular epidemiology of respiratory syncytial virus in children with acute respiratory illnesses in Africa: A systematic review and meta-analysis
Source: J Glob Health. 2023 Jan 14;13:04001. doi: 10.7189/jogh.13.04001 (PMC9840062; doi:10.7189/jogh.13.04001)
Supplement: Online Supplementary Document [file jogh-13-04001-s001.zip › Appendix S1 Online Supplementary Document.pdf]

Appendix S1 Online Supplementary Document

Molecular Epidemiology of Respiratory Syncytial Virus in Children with Acute Respiratory Illnesses in Africa: a Systematic Review and Meta-Analysis

Belay Tafa Regassa, Lami Abebe Gebrewold, Wagi Tosisa Mekuria and Nega Assefa Kassa

1. Search Terms for PubMed, Scopus, CINAHL and Global Index Medicus databases

	**Concept #1**	**Concept #2**	**Concept #3**
Free text word	Molecular epidemiology	Respiratory Syncytial Virus	Acute respiratory illnesses
MeSH Term	"Molecular Epidemiology"	"Respiratory Syncytial Viruses"<br>"Respiratory Syncytial Virus Infections"<br>"Respiratory Syncytial Virus, Human"	-
Related terms	Epidemiologic distribution	RSV	Acute respiratory illness*
	Epidemiology	Human respiratory syncytial virus	Respiratory illness*
	Prevalence	HRSV	Respiratory infection*
	Incidence	Respiratory syncytial virus A	Respiratory tract infection*
	Burden	Respiratory syncytial virus B	Upper respiratory tract infection*
	Magnitude	RSV-A	Lower respiratory tract infection*
	proportion	RSV-B	Acute upper respiratory tract infection*
			Acute lower respiratory tract infection*
			URTI
			AURI
			LRTI
			ALRI
			Respiratory disease*
			Respiratory disorder*

2. Search Strings used for different databases

Database	Search string
**PubMed**	("molecular epidemiology"[Title/Abstract] OR "Epidemiologic distribution"[Title/Abstract] OR "Epidemiology"[Title/Abstract] OR "prevalence"[Title/Abstract] OR "incidence"[Title/Abstract] OR "burden"[Title/Abstract] OR "magnitude"[Title/Abstract] OR "proportion"[Title/Abstract] OR "molecular epidemiology"[MeSH Terms]) AND ("Respiratory Syncytial Virus"[Title/Abstract] OR "RSV"[Title/Abstract] OR "Human respiratory syncytial virus"[Title/Abstract] OR "HRSV"[Title/Abstract] OR "RSV-A"[Title/Abstract] OR "RSV-B"[Title/Abstract] OR "Respiratory syncytial virus A"[Title/Abstract] OR "Respiratory syncytial virus B"[Title/Abstract] OR "Respiratory Syncytial Viruses"[MeSH Terms] OR "Respiratory Syncytial Virus Infections"[MeSH Terms] OR "respiratory syncytial virus, human"[MeSH Terms]) AND ("acute respiratory illness*"[Title/Abstract] OR "respiratory illness*"[Title/Abstract] OR "respiratory infection*"[Title/Abstract] OR "respiratory tract infection*"[Title/Abstract] OR "upper respiratory tract infection*"[Title/Abstract] OR "lower respiratory tract infection*"[Title/Abstract] OR "acute upper respiratory tract infection*"[Title/Abstract] OR "acute lower respiratory tract infection*"[Title/Abstract] OR "URTI"[Title/Abstract] OR "AURI"[Title/Abstract] OR "LRTI"[Title/Abstract] OR "ALRI"[Title/Abstract] OR "respiratory disease*"[Title/Abstract] OR "respiratory disorder*"[Title/Abstract])
**Scopus**	((TITLE-ABS-KEY (molecular AND epidemiology) OR TITLE-ABSKEY (epidemiologic AND distribution) OR TITLE-ABS-KEY (epidemiology) OR TITLE-ABSKEY (prevalence) OR TITLE-ABS-KEY (incidence) OR TITLE-ABS-KEY (burden) OR TITLE-ABSKEY (magnitude) OR TITLE-ABS-KEY (proportion))) AND (((TITLE-ABSKEY (respiratory AND syncytial AND virus)) OR (TITLE-ABSKEY (respiratory AND syncytial AND viruses)) OR (TITLE-ABSKEY (respiratory AND syncytial AND virus AND infections)) OR (TITLE-ABSKEY (respiratory AND syncytial AND virus, AND human)) OR (TITLE-ABS-KEY (rsv))) OR ((TITLE-ABSKEY (human AND respiratory AND syncytial AND virus) OR TITLE-ABS-KEY (hrsv) OR TITLE-ABSKEY (respiratory AND syncytial AND virus AND a) OR TITLE-ABSKEY (respiratory AND syncytial AND virus AND b) OR TITLE-ABS-KEY (rsv-a) OR TITLE-ABS-KEY (rsvb)))) AND ((TITLE-ABS-KEY (acute AND respiratory AND illness*) OR TITLE-ABSKEY (respiratory AND illness*) OR TITLE-ABS-KEY (respiratory AND infection*) OR TITLE-ABSKEY (respiratory AND tract AND infection*) OR TITLE-ABSKEY (upper AND respiratory AND tract AND infection*) OR TITLE-ABSKEY (lower AND respiratory AND tract AND infection*) OR TITLE-ABSKEY (acute AND upper AND respiratory AND tract AND infection*) OR TITLE-ABSKEY (acute AND lower AND respiratory AND tract AND infection*) OR TITLE-ABS-KEY (urti) OR TITLE-ABSKEY (auri) OR TITLE-ABS-KEY (lrti) OR TITLE-ABS-KEY (alri) OR TITLE-ABSKEY (respiratory AND disease*) OR TITLE-ABS-KEY (respiratory AND disorder*)))
**CINAHL**	((Molecular+epidemiology)+OR+(Epidemiologic+distribution)+OR+(Epidemiology)+OR+(Prevalence)+OR+(Incidence)+OR+(Burden)+OR+(Magnitude)+OR+(proportion))+AND+((Respiratory+Syncytial+Virus)+OR+(Respiratory+Syncytial+Viruses)+OR+(Respiratory+Syncytial+Virus+Infections)+OR+(Respiratory+Syncytial+Virus%2c+Human)+OR+(RSV)+OR+(Human+respiratory+syncytial+virus)+OR+(HRSV)+OR+(Respiratory+syncytial+virus+A)+OR+(Respiratory+syncytial+virus+B)+OR+(RSV-A)+OR+(RSV B))+AND+(((Acute+respiratory+illness*)+OR+(Respiratory+illness*)+OR+(Respiratory+infection*)+OR+(Respiratory+tract+infection*)+OR+(Upper+respiratory+tract+infection*)+OR+(Lower+respiratory+tract+infection*)+OR+(Acute+upper+respiratory+tract+infection*)+OR+(Acute+lower+respiratory+tract+infection*)+OR+(URTI)+OR+(AURI)+OR+(LRTI)+OR+(ALRI))+OR+((Respiratory+disease*)+OR+(Respiratory+disorder*)))
**Global Index Medicus**	tw:((tw:(tw:((tw:(molecular epidemiology)) OR (tw:(epidemiologic distribution)) OR (tw:(epidemiology)) OR (tw:(prevalence)) OR (tw:(incidence)) OR (tw:(burden)) OR (tw:(magnitude)) OR (tw:(proportion))))) AND (tw:(tw:((tw:(respiratory syncytial virus)) OR (tw:(respiratory syncytial viruses)) OR (tw:(respiratory syncytial virus infections)) OR (tw:(respiratory syncytial virus, human)) OR (tw:(rsv)) OR (tw:(human respiratory syncytial virus)) OR (tw:(hrsv)) OR (tw:(respiratory syncytial virus a)) OR (tw:(respiratory syncytial virus b)) OR (tw:(rsv-a)) OR (tw:(rsv-b))))) AND (tw:(tw:((tw:(acute respiratory illness*)) OR (tw:(respiratory illness*)) OR (tw:(respiratory infection*)) OR (tw:(respiratory tract infection*)) OR (tw:(upper respiratory tract infection*)) OR (tw:(lower respiratory tract infection*)) OR (tw:(acute upper respiratory tract infection*)) OR (tw:(acute lower respiratory tract infection*)) OR (tw:(urti)) OR (tw:(auri)) OR (tw:(lrti)) OR (tw:(alri)) OR (tw:(respiratory disease*)) OR (tw:(respiratory disorder*))))))
